# WORDS OF ADVICE FOR RESEARCHERS: A QUALITATIVE STUDY ON PEOPLE LIVING WITH DEMENTIA’S EXPERIENCES IN RESEARCH

**DOI:** 10.1093/geroni/igad104.0284

**Published:** 2023-12-21

**Authors:** Ha Neul Kim, Jen Hirsch

**Affiliations:** Michigan State University, East Lansing, Michigan, United States; Michigan State University, East Lansing, Michigan, United States

## Abstract

People living with dementia are the focus of much research, but little is known about their research experiences and how they perceive the world of research. This qualitative study examined the motivations, experiences, and words of advice from people living with dementia regarding their research experiences including both social research and clinical trials. Semi-structured interviews were conducted individually with 7 people, each was interviewed twice for about an hour. Prior to the interview, participants were screened for decisional capacity using a modified UBACC (University of California, San Diego Brief Assessment of Capacity to Consent) to make sure of their understanding of the project. After participants passed the screen, they provided verbal consent. Thematic analysis was conducted to analyze interviews and draw out meaningful themes. Five primary themes emerged: 1) the importance of people living with dementia and their communities in research recruitment, 2) the need for diversity and inclusion in dementia research, 3) the need for a positive attitude of researchers, 4) motivation focused on making contributions and finding a cure for oneself, and 5) the need for balance between showing expertise and explaining with less jargon. The study indicates that there are improvements researchers can make in terms of inclusiveness, listening to people’s voices, and engaging people with lived experiences in the research design process, which inspires more participatory research in the dementia research field. This study was supported by the Corey Endowment Award; and the Research Scholars Award.

